# Comparison of Gut Microbiota Between Golden and Brown Noble Scallop *Chlamys nobilis* and Its Association With Carotenoids

**DOI:** 10.3389/fmicb.2020.00036

**Published:** 2020-02-07

**Authors:** Hongxing Liu, Kar Soon Tan, Xinxu Zhang, Hongkuan Zhang, Dewei Cheng, Ye Ting, Shengkang Li, Hongyu Ma, Huaiping Zheng

**Affiliations:** ^1^Key Laboratory of Marine Biotechnology of Guangdong Province, Institute of Marine Sciences, Shantou University, Shantou, China; ^2^Mariculture Research Center for Subtropical Shellfish & Algae of Guangdong Province, Shantou, China; ^3^STU-UMT Joint Shellfish Research Laboratory, Shantou University, Shantou, China; ^4^Institute for Advanced Study, Shenzhen University, Shenzhen, China

**Keywords:** scallop, *Chlamys nobilis*, carotenoids, gut microbe, *Brevundimonas*, bacterial abundance

## Abstract

Many marine bivalves are regarded as healthy foods due to their high carotenoid content. Only plants and microorganisms have natural carotenoids biosynthesis ability, hence, animals such as bivalves must obtain carotenoids from their diets. Due to the filter-feeding behavior of bivalves, they have high diversity of gut microbes. However, the relationship between gut microbes and carotenoids has not been explored in mollusks. In the present study, the interaction between gut microbes and carotenoids in two polymorphic noble scallop *Chlamys nobilis*, golden scallops (designated GG) and brown scallops (designated BW), were studied. The gut of GG and BW showed statistically different bacteria communities. Results from 16S rRNA gene sequencing and qPCR analysis revealed that the gut of GG had significantly higher relative abundance of carotenoids-producing bacteria *Brevundimonas*, compared with BW. Moreover, HPLC-MS analysis showed that isolate *Brevundimonas* could produce astaxanthin. The current findings are very useful as they could form the basis for future studies in determining the relationship between gut microbiota and carotenoids absorption in bivalves.

## Introduction

Gut microbiota is regarded as an integral component of the host because of its important role in host digestion and health (Sommer and Bäckhed, 2013; Sonnenburg and Bäckhed, 2016). Studies in humans and other vertebrates have revealed that gut microbiota produces many metabolites, including short-chain fatty acids (Immerseel et al., 2010), essential vitamins and amino acids (Ikeda et al., 1979; Ramotar et al., 1984; Lozupone et al., 2012; Mardinoglu et al., 2015), as well as some digestive enzymes (Yolton and Savage, 1976; Dabek et al., 2008; Carmody and Turnbaugh, 2014) that are beneficial to the host. The microbiomes of terrestrial invertebrates, especially insects such as wasps (Adams et al., 2011), flies (Erkosar et al., 2013), termites (Poulsen et al., 2014), and beetles (Salem et al., 2017), have been studied and shown to be involved in host nutrient acquisition provided by symbiotic bacteria. On the other hand, in marine invertebrates, such as mollusks (Trabal et al., 2014), crustaceans (Givens et al., 2013) and sponges (Richardson et al., 2012; Schmitt et al., 2012), microbiome studies have mostly focused on the dynamics of bacterial communities under various nutrients and environmental conditions. A growing number of studies have shown that symbiotic bacteria provide carotenoids to many invertebrates, as in the accessory nidamental glands (ANG)-bacterial community in squids (Barbieri et al., 1996; Grigioni et al., 2000) and endosymbiotic bacteria in whiteflies (Sloan and Moran, 2011).

Carotenoids are lipid-soluble tetraterpenoid compounds biosynthesized by plants and microorganisms (algae, bacteria, and fungi). In general, animals cannot biosynthesize carotenoids and must, therefore, obtain them from their diets (Tanumihardjo, 2013). However, in recent years, some insects such as aphids (Moran and Jarvik, 2010; Nováková and Moran, 2011), gall midges (Cobbs et al., 2013), and spider mites (Altincicek et al., 2012) have been found to be able to synthesize their own carotenoids by *trans*-kingdom transfer genes from fungi. As important antioxidants, carotenoids play many important functions in human and animal health (Tanumihardjo, 2013). Moreover, as natural pigments (with color ranging from yellow to red), carotenoids are also involved in the body coloration of animals (Maoka, 2009), and have been reported in vertebrates such as fish (Wang et al., 2006), frogs (Umbers et al., 2016), and birds (Negro and Garrido-Fernèndez, 2000; Weaver et al., 2018), as well as invertebrates such as silkworm *Bombyx mori* (Tabunoki et al., 2004), sea urchin *Strongylocentrotus intermedius* (Borisovets et al., 2002) and turban shell *Turbo cornutus* (Maoka, 2011).

In mollusks, which are the second largest phylum in the animal kingdom, some groups including Polyplacophora, Gastropods, Bivalves, and Cephalopods are rich in carotenoids ranging from 10 to 140 μg/100 g (Matsuno and Hirao, 1989), albeit varying in different tissues (Kantha, 1989). Moreover, mollusks contain a wide range of carotenoids such as β-carotene, lutein, zeaxanthin, diatoxanthin, and astaxanthin, which is mainly obtained from microalgae, with some metabolically modified into other carotenoid forms (Matsuno, 2001). Some mollusks are therefore important sources of carotenoids for humans.

The noble scallop *Chlamys nobilis* is a marine bivalve, which is very popular in China, and naturally distributed in Vietnam, China, and Japan. Noble scallops display polymorphism in their shell colors (i.e., golden, purple, yellow, brown, and white) and muscles color (golden or white). Our previous studies have shown that the golden scallops, which have golden shells and adductor, are very rich in total carotenoid content compared with brown scallops with brown shells and white adductors (Zheng et al., 2010). The *SRB-like-3* gene is believed to be involved in the accumulation of carotenoids in golden scallops (Liu et al., 2015). Intriguingly, we also observed that golden scallops have a golden gut, while brown scallops have a white gut. Therefore, we wondered whether the golden gut of golden scallops had a higher carotenoid content than white guts and, if so, whether there could be some specific gut bacteria associated with this pigmentation and carotenoid content.

In the present study, two noble scallops *C. nobilis* from the same breeding stock. i.e., golden scallops (with golden shells and golden gut, designated GG) and brown scallops (with brown shells and white gut, designated BW), were used. The total carotenoids content (TCC) in the adductor, hemolymph, and gut were compared, while the bacterial 16S rRNA genes in the gut were sequenced and analyzed. Finally, some carotenoids-producing bacteria were isolated by culture-based methods and identified using high performance liquid chromatography–mass spectrometry (HPLC-MS).

## Materials and Methods

### Experimental Materials and Sample Collection

**FIGURE 1:**
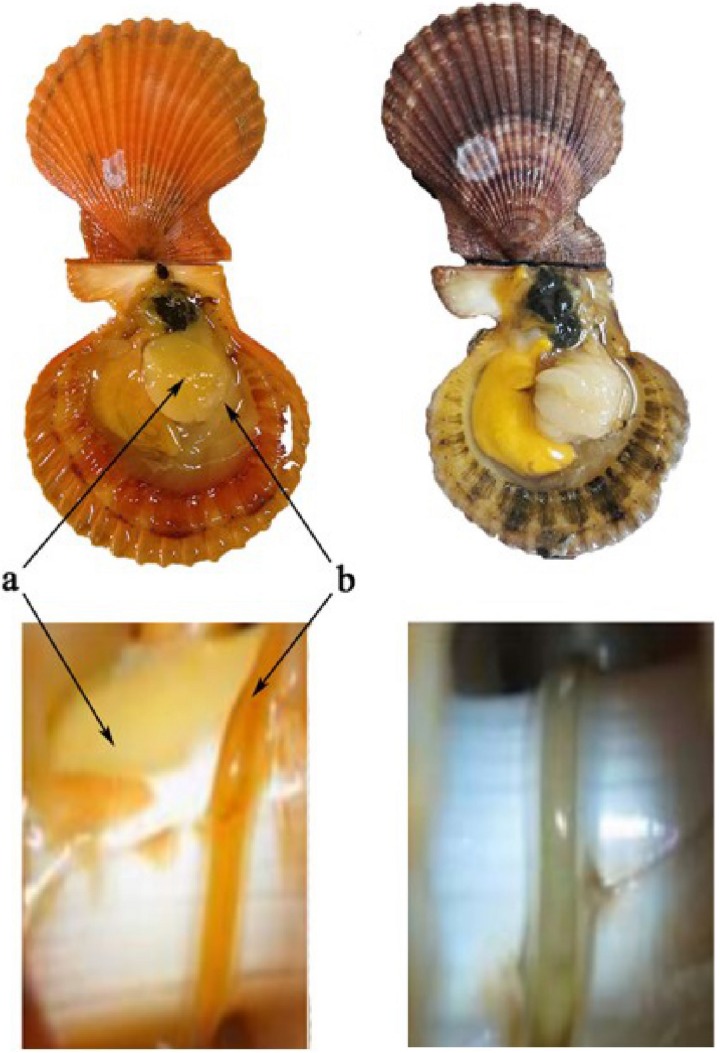
Golden and brown noble scallops and their guts. The golden scallop with golden shell, golden adductor and golden gut (left, named as GG), and the brown scallop with orange shell, white adductor and white gut (right, named as BW). Two kinds of tissues: adductor (a) and gut (b).

Golden and brown noble scallop strains originating from the same family with the same parents, established using the artificial breeding method previously described by Zheng et al. (2013), were cultured under the same conditions from D-shape larvae to adults at Shen’ao Bay (23°29′N, 117°07′E) of Guangdong Province, China. One hundred individuals of 1-year-old adult female scallops (50 golden and 50 brown individuals) were randomly selected from the two strains for this experiment (Figure 1). After cleaning, individual scallops were measured, i.e., shell length with a vernier caliper to the nearest 0.1 mm and weighed on a digital balance to the nearest 0.1 g. The scallops were then placed in two 500 L polyethylene tanks for 1 week for acclimation to laboratory conditions. Each scallop was fed daily with 4 × 10^6^ cells of *Nitzschia closterium*. The seawater temperature was 23.6°C and salinity 31.1 psu, with water changed daily.

Five scallops of approximately the same size (the same shell length) were randomly selected from each tank. The outer surface of each scallop’s gut was washed thoroughly with sterile water and disinfected with 75% ethanol before being dissected. Next, each gut was placed into a sterile tube, and fully homogenized with 1 mL sterile sea water using a sterile plastic pestle. Samples were then placed into two eppendorf tubes, for bacterial DNA extraction and to isolate bacteria for culture. The gut, adductor and hemolymph of five other scallops from each tank were sampled according to the method described previous (Zheng et al., 2012a) for TCC determination. All samples were stored in liquid nitrogen until processing, except the gut samples used for bacteria isolation.

### Determination of Total Carotenoids Content (TCC)

Total carotenoids content was determined as described previously (Zheng et al., 2010). Briefly, the adductor, gut and hemolymph samples were freeze-dried in a vacuum pump (ScanVac CoolSafe, LaboGene, Denmark) for 48 h, and ground to homogenous powder in a mortar. Next, 0.02 g of each sample was mixed with 1 mL acetone with shaking (200 rpm) for 1 h at 25°C in the dark. The extract was centrifuged at 5000 rpm for 5 min. The supernatant was measured with a spectrophotometer (UV2501PC, SHIMADZU, Japan) from 400 to 700 nm wave length. The TCC (μg/g dry weight) was calculated by using the extinction coefficient E (1%, 1 cm) of 1.900 at the absorption value of 480 nm (Yanar et al., 2004).

### DNA Extraction, PCR Amplification and Illumina Sequencing

The gut bacterial DNA was extracted using the QIAamp DNA stool kit (Qiagen) following the manufacturer’s instructions, and the concentration and quality of the DNA determined at A260 and A280 nm using an ND-1000 spectrophotometer (NanoDrop Technologies, Wilmington, DE, United States) and 1% agarose gel electrophoresis with Gel Red^TM^ staining. The bacterial 16S rRNA gene was amplified by PCR (Caporaso et al., 2011) using the primers 515F (5′-GTGCCAGCMGCCGCGG-3′) and 806R (5′-GGACTACHVGGGTWTCTAAT-3′), which target the hypervariable V4 region of the 16S rRNA gene. Unique eight-base barcodes were added to each primer to distinguish the PCR products. The PCR product was gel purified using the QIAquick Gel Extraction Kit (Qiagen, Germany), and 30 ng of each purified PCR product was subjected to sequencing using the Illumina HiSeq 2000 platform (Total Genomics Solution Institute, Shenzhen, China). Raw reads containing a 50 bp continuous fragment or with an average quality score less than 30 and/or any ambiguities were removed. Reads containing more than two mismatches to the primers or more than one mismatch to the barcode were discarded. Filtered reads were then merged by overlapping paired-end reads using FLASH (Version1.2.7) with an overlap length of more than 15 bp.

### Microbial Community Compositions and Their Potential Functions

Operational Taxonomic Units (OTUs) were classified using USEARCH (v7.0.1090) (Wang et al., 2007) at a 97% similarity level. The taxonomy assignment of each 16S rRNA gene sequence was analyzed by the RDP Classifier^1^ using assign_taxonomy.py in QIIME. The relative abundance of each OTU was examined at different taxonomic levels. Alpha diversity indices (Shannon and Chao 1) were calculated by alpha_diversity.py in QIIME. Alpha diversity indices were compared among two groups with a non-parametric *t*-test (Monte Carlo, 999 permutations) in QIIME (Schloss et al., 2009). The principal coordinate analysis (PCoA) based on weighted UniFrac distance matrix was performed in QIIME. Heatmap and Venn diagrams of gut microbiota were generated from the R project for statistical computing^2^ To explore the potential functions of the bacterial communities, the PICRUSt (Phylogenetic Investigation of Communities by Reconstruction of Unobserved States) was used to predict the metabolic pathways in the gut of each sample (Langille et al., 2013). OTUs data were mapped to the Greengenes database and 81% of genes were classified to a Tier 1 KEGG orthology (KO) function. The nearest sequenced taxon index (NSTI) scores averaged 0.053 ± 0.012, indicating a relatively good match to reference genomes (ideal NSTI ≤ 0.03). Sequenced data has been deposited at NCBI under Bioproject number PRJNA574069.

### Isolation of Carotenoid-Producing Bacteria

Three solid media [Nutrient agar, Luria-Bertani (LB) agar, and Marine agar 2216] were used to isolate carotenoid-producing bacteria (Asker et al., 2007). The gut samples of GG (*n* = 5) were used as the source of bacteria. Samples were homogenized with sterile sea water using a sterile plastic pestle. Next, 0.2 ml of a 10-fold dilution of each sample was spread on LB solid medium in duplicate, and the plates incubated at room temperature.

After incubation for 48 h, yellow-, orange-, or red-colored bacterial colonies were isolated and identified by 16S rRNA gene-based analysis using the universal bacterial primers 27F and 1492R (Lane, 1991). Isolates that shared 99% or higher 16S rRNA gene sequence similarity were considered as the same species. To identify carotenoid-producing bacteria, the carotenoids of bacterial cells were extracted by organic solvent (acetone). The absorbance of the extract was assessed at λ between 260 and 700 nm at room temperature by using a spectrophotometer (BioTek, United States). The potential carotenoid producing bacteria are currently preserved in our laboratory at Shantou University. Further identification of bacterial carotenoids was carried out using HPLC-MS system (Agilent 1100, United States) according to the method described by Asker et al. (2007). Astaxanthin (Sigma, St. Louis, MO, United States) was used as an external standard.

### Quantitative PCR

Quantitative PCR was performed to quantify the abundance of 16S rRNA gene in gut samples of scallops. Total gut bacteria were quantified by real-time PCR using the 16S RNA gene-specific primers (Guo et al., 2008). Species specific real-time PCR were applied to quantify the *Brevundimonas* 16S rRNA gene located in the V4–V5 hypervariable region and normalized by real-time PCR data of the host β*-actin* gene (Table 1). The quantitative real-time PCR was performed on an ABI 7300 Real-Time Detection System (Applied Biosystems, United States) using the SYBR Premix Ex Taq II qRT-PCR Kit (TaKaRa). PCR cycle threshold (Ct) values and standard curve were obtained by amplifying 10-fold serially diluted plasmids. Each amplification efficiency was also determined.

**TABLE 1:** Primer utilized for gene analysis.

Primer name	Sequence (5′–3′)	Application	References
16S-QF	ACTCCTACGGGAGGCAGCAG	qPCR of V4 hypervariable region of bacterial 16S rRNA gene	Guo et al., 2008
16S-QR	ATTACCGCGGCTGCTGG		
β-actinF	CAAACAGCAGCCTCCTCGTCAT	qPCR of host β*-actin* gene	Zheng et al., 2012b
β-actinR	CTGGGCACCTGAACCTTTCGTT		
16S-27F	AGAGTTTGATCCTGGCTCAG	PCR of bacterial 16S rRNA gene	Lane, 1991
16S-1492R	GGTTACCTTGTTACGACTT		
Bre-QF	GCCTTCGCCACTGGTGTTCTTCC	qPCR of V4-V5 hypervariable region of *Brevundimonas* 16S rRNA gene	This study
Bre-QR	TGCCTTTGATACTGGGTGTCTTG		

### Statistical Analysis

Total carotenoids content, relative abundance of bacterial genera and relative abundance of functional gene are expressed as mean ± standard deviation of the mean (SD).

The abundance of bacterial genera and functional gene with statistically significant difference were assessed using Metastats (White et al., 2009). Metastats data were then evaluated using the non-parametric Kruskal–Wallis test with false discovery rate (FDR) correction for multiple testing and significance for all analyses was set at *P* < 0.05 unless noted otherwise.

## Results

### Comparison of Total Carotenoids Content (TCC) in Tissues of Scallops

**FIGURE 2:**
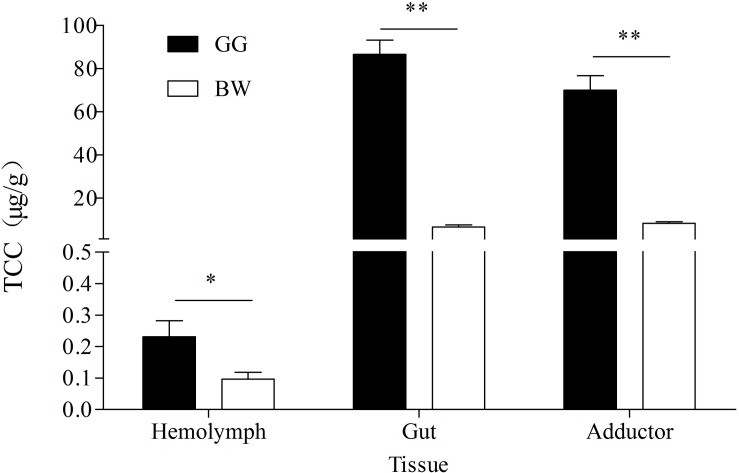
TCC in hemolymph, gut and adductor of golden and brown noble scallops. Date are shown as the mean ± SD (*n* = 5). ^∗^*P* < 0.05; ^∗∗^*P* < 0.01 (*t*-test).

The TCC from three tissues of golden and brown scallops is shown in Figure 2. In all tested tissues, GG had significantly higher TCC compared with BW (*P* < 0.05). Gut had the highest TCC among the three tissues tested.

### Comparison of Gut Bacterial Diversity Between Golden and Brown Scallops

After filtering out low quality reads, a total of 365360 reads of the V4 region of the 16S rRNA genes were obtained from 10 samples. Reads were clustered into OTUs at a 97% similarity level, in which each OTU represented a unique phenotype. A total of 1862 unique OTUs were obtained from gut bacterial community. Over 54% of all OTUs were shared by the gut of the two scallops, with GG having more unique OTUs compared with BW (data no shown).

### Comparison of Gut Bacterial Composition Between Golden and Brown Scallops

**FIGURE 3:**
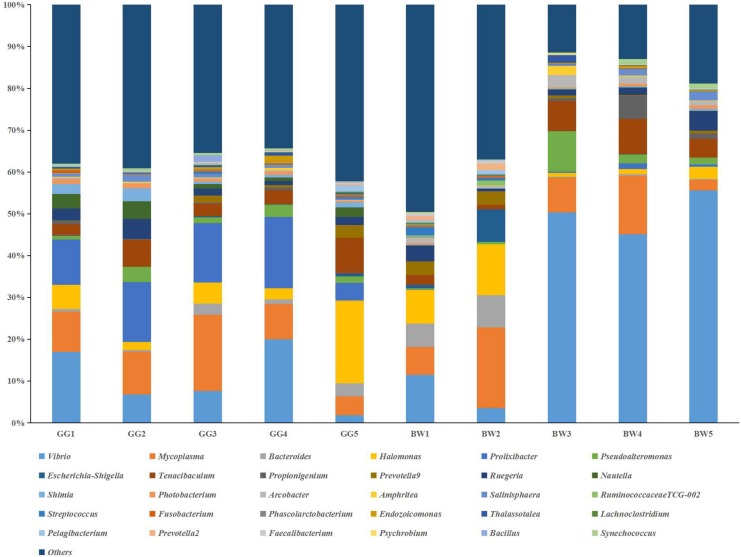
Relative abundance of the genus level in guts of noble scallops. Sample from GG1 to GG5 was collected from GG, and sample from BW1 to BW5 was collected from BW, respectively. Groups within the same bacterial genus are indicated by different shades of the same color.

**FIGURE 4:**
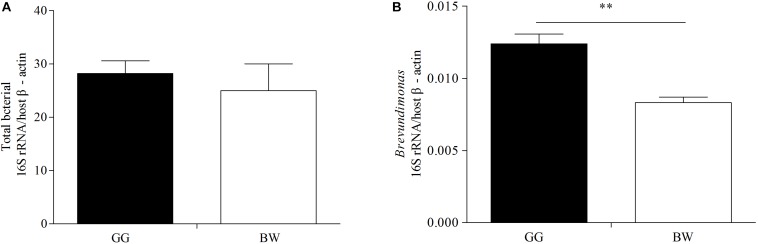
Quantification the 16S rRNA gene of total bacteria **(A)** and *Brevundimonas* **(B)** in gut samples from two groups (GG and BW). The levels of total bacterial 16S rRNA gene and *Brevundimonas* 16S rRNA gene were normalized by the expression of the host β-actin gene. The values are expressed as means ± SD (*n* = 5). The asterisks (^∗∗^ *P* < 0.01) indicate significant differences in two groups.

The bacterial profiles of the 10 samples analyzed generated 37 distinctive bacterial phyla. Both GG and BW had similar gut bacterial composition at the phylum level. The dominant phyla include Proteobacteria (39.3% ∼ 84.1%), Tenericutes (3.4% ∼ 19.9%), Bacteroidetes (7.1% ∼ 32.6%), Firmicutes (0.4% ∼ 18.9%), Fusobacteria (0.2% ∼ 10.0%), and Actinobacteria (0.1% ∼ 1.5%), which accounted for 86.78–98.67% of the total reads. At the genus level (Figure 3), *Vibrio* (3.5% ∼ 71.1%), *Mycoplasma* (2.6% ∼ 55.4%) and *Bacteroides* (0.05% ∼ 20.1%) were dominant in all 10 libraries. GG had significantly lower relative abundance of *Vibrio* (average 12.1%) compared with BW (average 30.7%) (*P* < 0.05). However, there was no significant difference in the abundance of the gut bacterial 16S rRNA gene between GG and BW (Figure 4A).

### Comparison of Bacterial Community Structure Between Golden and Brown Scallops

**FIGURE 5:**
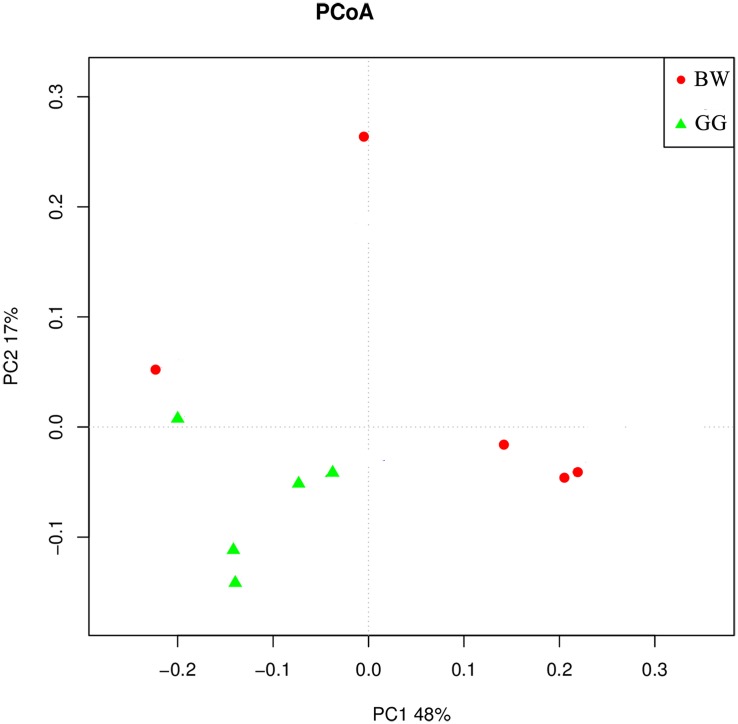
The weighted UniFrac PCoA analysis of the gut microbiota of noble scallops. The GG samples are included in green triangle, and the BW samples are included in red circle. PC1 and PC2 are shown on the *x*-axis and *y*-axis, respectively. The percentage of variance explained by each coordinate is shown.

The composition of bacterial community measured by the weighted UniFrac PCoA analysis is illustrated in Figure 5. The GG samples clustered together, whereas the BW samples were more variable and dispersed. In addition, the ANOSIM test results (*R* = 0.829, *P* = 0.024) indicated significant differences between the bacterial communities of GG and BW.

### Comparison of Biosynthetic Capabilities of Gut Microbiota From Golden and Brown Scallops

**FIGURE 6:**
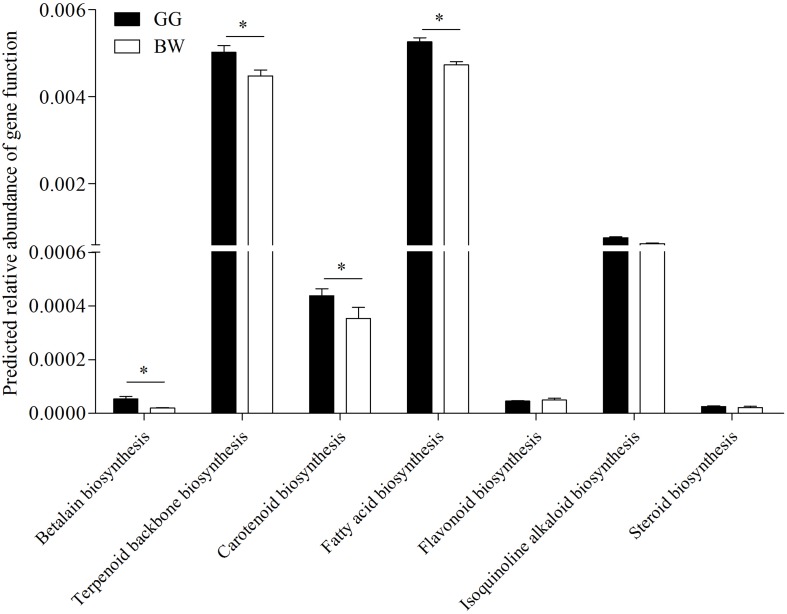
Predicted relative abundance of partial functional genes of PICRUSt-generated functional profile of gut microbiota between golden and brown noble scallops. Significant differences in gene categories related to KEGG pathways at level 3 between two scallop groups (GG and BW). Date are shown as the mean ± SD ^∗^ *P* < 0.05.

In order to estimate the bacteria biosynthetic abilities, PICRUSt was used to predict the gut microbiome functions. As shown in Figure 6, compared with brown scallops (BW) the gut microbiota of golden scallops (GG) contained higher abundance of functional genes including those involved in the biosynthesis of carotenoids, fatty acids, terpenoids backbone, and betalain at KEGG level 3 (*P* < 0.05).

### Isolation and Identification of Potential Carotenoids-Producing Bacteria in Gut

A total of seven potential carotenoids-producing bacteria were isolated from the gut of golden scallops and their phylogenetic affiliation was studied by a conventional 16S rRNA gene-based method. As shown in Table 2, the potential carotenoids-producing bacteria were classified into four genera (*Brevundimonas*, *Sphingomonas*, *Rhodococcus*, and *Acinetobacter*). Most isolates were obtained from LB medium, possibly due to its relatively high salt concentration (1% NaCl). All the potential carotenoid-producing strains (G2, G3, G14, G17, G36, G42, G61) produced yellow, orange or red pigments, and their extracts have similar light absorbance (around 480 nm) with carotenoids.

**TABLE 2:** Phylogenetic and physiological characteristics of the potential carotenoids-producing strains.

Strains number	Isolated media^A^	Closest GeneBank relative (accession number)	Similarity (%)	Query length (bp)	Colony^B^	Related OTUs^C^	Relative abundance^D^		Carotenoid spectrum (λmax nm)
								
							GG	BW	
G2	L	*Brevundimonas vesicularis* (NZ_CP022048.2)	99	1392	R/s/g	OTU_124	0.096 ± 0.012^a^	0.02 ± 0.009^b^	476
G3	L	*Brevundimonas subvibrioides* (NC_014375.1)	97	1395	R/g/s/S	OTU_124	0.096 ± 0.012^a^	0.02 ± 0.009^b^	476, 484
G14	L	**Sphingomonas melonis** TY (NZ_CP017578.1)	96	1386	Y/S	OTU_52	0.22 ± 0.14^a^	0.17 ± 0.17^a^	442
G17	L/N	**Sphingomonas paucimobilis** NBRC 13935 (NZ_BBJS01000072.1)	99	1401	Y/s/L	OTU_52	0.22 ± 0.14^a^	0.17 ± 0.17^a^	472
G36	L/N	*Rhodococcus erythropolis* CCM2595 (NC_022115.1)	99	1411	R/L	OTU_1020	0.01 ± 0.023^a^	0.018 ± 0.031^a^	449, 492
G42	L/N	*Rhodococcus erythropolis* CCM2595 (NC_022115.1)	98	1427	R/S	OTU_1020	0.01 ± 0.023^a^	0.018 ± 0.031^a^	452
G61	N/M	*Acinetobacter pittii* PHEA-2 (NC_016603.1)	99	1442	O/L	OTU_30	0.43 ± 0.11^a^	0.41 ± 0.21^a^	478

*^*A*^N, Nutrient agar, L, LB agar, M, Marine agar 2216. ^*B*^Y, yellow, O, orange, R, red, s, shiny, g, gummy, L, large, S, small. ^*C*^The related sequences of OTUs share the highest similar with the related 16S rDNA gene sequences of isolates. ^*D*^The relative abundance (%) of related OTUs was expressed as the mean ± SD (*n* = 5). Different letters denote significant differences between two groups within same strains. Significance was calculated using the Kruskal–Wallis test with false discovery rate (FDR) correction.*

### Abundance of Carotenoids-Producing Bacteria *Brevundimonas* in the Gut of Golden and Brown Scallops

Based on the result of 16S rRNA high-throughput sequencing, we found that the abundance of the genera *Brevundimonas* (OTU_124 corresponding to strains G2 and G3) was significantly higher (*P* < 0.05) in the gut of GG compared with BW (Table 2). Similar results were obtained by quantitative PCR analysis, where the abundance of *Brevundimonas* 16S rRNA gene was significantly higher in GG compared with BW (Figure 4B). However, the abundance of other potential carotenoids-producing bacteria (*Sphingomonas*, *Rhodococcus*, and *Acinetobacter*) was not statistically different between two groups (Table 2).

**FIGURE 7:**
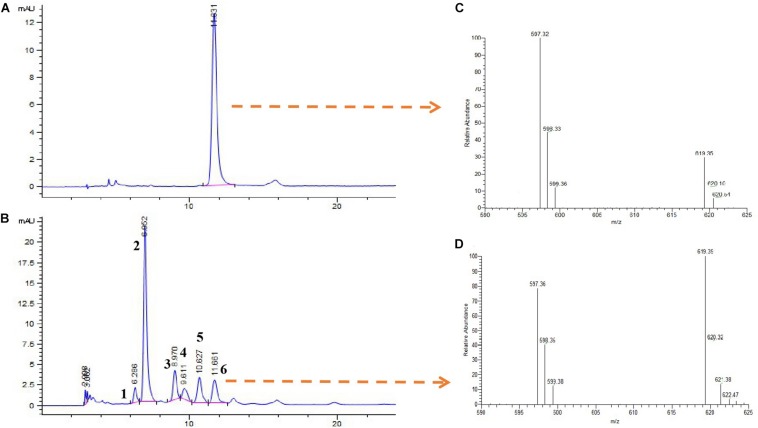
High-performance liquid chromatography and mass spectra (HPLC-MS) analysis of carotenoid extract from the isolate *Brevundimonas*. HPLC elution profiles (λmax 480 nm) of astaxanthin **(A)** and carotenoids produced by the isolate *Brevundimonas* **(B)**. Mass spectra of **(C)** astaxanthin standard and **(D)** astaxanthin produced by the isolate *Brevundimonas* (peak 6).

In addition, the extracts of the isolate *Brevundimonas* were identified by HPLC-MS (Figure 7). The results showed that the *Brevundimonas* produced six types of carotenoids with peaks (1, 2, 3, 4, 5, and 6) recorded at different *R*_*t*_. The *m*/*z* of peak 6 was 597 [M^+^ + 1 and M^+^ + 23], which is similar to the astaxanthin standard (Figure 7).

## Discussion

A broad range of studies on animal-associated bacteria communities have advanced our understanding of the interaction between animals and bacteria. Gut microbiota plays important roles in the health, physiology, and behavior of animals (Erkosar et al., 2013; Mardinoglu et al., 2015). Although gut microbiota has been extensively explored in vertebrates, gut microbiota of marine invertebrates is less understood. In the present study, we compared the TCC and gut microbiota between golden and brown scallops. The golden and brown scallops used in the present study were cultivated under the same environmental conditions (on a farm in Nan’ao Island) and fed on the same microalgae composition. Therefore, we assume that the different levels of accumulated carotenoids between golden and brown scallops were contributed to by their gut microbiota. Based on the 16S rRNA gene high-throughput sequencing, GG had different bacteria communities compared with BW. Among the three major genera identified (*Vibrio*, *Mycoplasma*, and *Bacteroides*), only the relative abundance of *Vibrio* was statistically different, with the relative abundance of *Vibro* in the gut of GG significantly lower than that of BW. As a potential pathogenic bacterial, *Vibrio* might be associated with the immunity of bivalve mollusks (Pierce et al., 2016), suggesting that a high carotenoid content in the gut of GG might help lower the relative abundance of *Vibrio*. In addition, *Mycoplasma* and *Bacteroides* were the dominant bacteria in gut of noble scallops, suggesting that these bacterial genera were well-adapted to conditions of the scallops’ gut. Recent studies have shown that *Bacteroides* are able to digest marine carbohydrates (Sonnenburg et al., 2010; Hehemann et al., 2012). Similarly, previous studies have indicated that *Mycoplasma* was the dominant bacteria in the gut of aquatic animals, but their function remains unknown (Bano et al., 2007).

Our previous studies show that the adductor and mantle of golden scallops contained significantly higher carotenoids content compared with brown scallops, with these differences in carotenoids content attributed to genetic factors (Zheng et al., 2013) as well as control by carotenoid absorption-related genes such as *SRB-like-3* (Liu et al., 2015). In the present study, golden scallops were found to contain significantly higher carotenoids content in their adductor, guts and hemolymph compared with brown scallops. It is believed that carotenoids in diets are absorbed by the gut and transported in hemolymph to be accumulated in other body tissues (Liu et al., 2015).

One notable and interesting observation in that current study was that some carotenoid-producing bacteria that could contribute to the accumulation of carotenoids in golden scallops were identified. Similar observations have been documented in cephalopods (Barbieri et al., 1996; Grigioni et al., 2000) and sponges (Miki et al., 1994), where gut bacteria were suggested to provide carotenoids to their host. Analysis of the potential functions of bacterial communities revealed that the gut microbiota in GG had a high abundance of functional genes that are involved in the biosynthesis of fatty acids, terpenoids backbone and carotenoids, which could partly explain the gold coloration and deposition of carotenoids in the gut of golden scallops. This observation is consistent with Zheng et al. (2012a), who reported a strong positive correlation between TCC and total lipid content in tissues of noble scallops. Moreover, the terpenoids backbone is an essential precursor for the biosynthesis of carotenoids (Römer and Fraser, 2005; Yonekura and Nagao, 2010). The 16S rRNA high-throughput sequencing results also revealed 11 bacteria genera that are potential carotenoid producers (Supplementary Table S1), although these bacteria constituted a small group (<1%). Thus, in order to verify the occurrence of carotenoid-producing bacteria in the gut of noble scallops, a culture method was used. Indeed, a number of potential carotenoid-producing bacteria in the gut of GG were found, with phylogenetic analysis revealing that these bacteria belong to the genera *Brevundimonas*, *Sphingomonas*, *Rhodococcus*, and *Acinetobacter*. A high proportion of the isolates comprised of *Brevundimonas* and *Sphingomonas*, which are well-known in carotenoid production (Asker et al., 2018). Similarly, *Rhodococcus* are a class of photosynthetic bacteria known to produce carotenoids such as Canthaxanthin (Hannibal et al., 2000). Although there is no information on carotenoid production by *Acinetobacter*, our results showed that some strains that belong to *Acinetobacter* exhibit orange pigmentation that has similar light absorbance with carotenoids (Table 2).

Intriguingly, our results revealed that the abundance of the carotenoid-producing bacteria *Brevundimonas* was significantly higher in the gut of GG compared with BW, although *Brevundimonas* only accounted for 0.03–0.05% of the total bacteria community in all tested individuals. These observations suggest that *Brevundimonas* are resident bacteria in the gut of noble scallops. To confirm the carotenoid biosynthetic ability of *Brevundimonas*, carotenoids were extracted from this isolate and identified using HPLC-MS. The data revealed that *Brevundimonas* could produce astaxanthin and other unknown carotenoids. Marine *Brevundimonas* have been reported to produce many carotenoids such as xanthophyll (Nishida et al., 2005), astaxanthin, adonixanthin, and other ketocarotenoids (Asker et al., 2018). Other studies including Teramoto et al. (2003), Stafsnes et al. (2010), and Asker et al. (2018) have all reported that marine bacteria are important sources of natural carotenoids. Therefore, we strongly believe that the high carotenoid content in the gut of golden scallops might be related to the carotenoid-producing bacteria *Brevundimonas*. Our results are also backed by similar previous studies, where isolated *Brevundimonas* sp. SD-212 was reported to produce several carotenoid compounds, including 2-hydroxyastaxanthin, 2-hydroxyadonixanthin, erythroxanthin, 2,2′-dihydroxyastaxanthin, 2,2′-dihydroxyadonixanthin, adonix anthin, and astaxanthin (Yokoyama et al., 1996). Thus, it seems that the other unknown carotenoids produced by isolate *Brevundimonas* in our current study could be hydroxylated derivatives of astaxanthin such as 2-hydroxyastaxanthin and 2,2′-dihydroxyastaxanthin. After all, astaxanthin is widely distributed in tissues of marine animals, including marine scallops (Suhnel et al., 2009). However, since the present study was not designed to assess the role of carotenoid producing bacteria in carotenoid accumulation in scallops, we are only able to speculate that some of the carotenoids absorbed by noble scallops could possibly originate from gut bacteria. Future studies would explore if some of the carotenoids absorbed by bivalves are from their gut bacteria.

## Conclusion

In conclusion, the gut of golden noble scallops contained significantly higher TCC compared with brown scallops. The gut of golden scallops has a different bacteria community and has a higher proportion of carotenoid-producing bacteria *Brevundimonas* compared with brown scallops. These observations are first observed in mollusks, and therefore provide valuable information for future studies on exploring carotenoids accumulation in marine invertebrates.

## Data Availability Statement

Sequence data has been deposited at NCBI under BioProject number PRJNA574069.

## Author Contributions

HL, HM, SL, and HuZ conceived and designed the experiments. KT, XZ, HL, and HuZ wrote the manuscript. HoZ, DC, and YT collected and prepared the samples.

## Conflict of Interest

The authors declare that the research was conducted in the absence of any commercial or financial relationships that could be construed as a potential conflict of interest.

## Footnotes

1 http://rdp.cme.msu.edu/

2 http://www.r-project.org/
