# Supplementary material for: Comparison of Gut Microbiota Between Golden and Brown Noble Scallop Chlamys nobilis and Its Association With Carotenoids
Source: Front Microbiol. 2020 Feb 7;11:36. doi: 10.3389/fmicb.2020.00036 (PMC7018768; doi:10.3389/fmicb.2020.00036)
Supplement: Supplementary file 1 [file Table_1.DOC]

| OTU numbers | Taxonomy | Relative abundance (%) | | | | | | | | | | *P* value |
| --- | --- | --- | --- | --- | --- | --- | --- | --- | --- | --- | --- | --- |
|  |  | GG1 | GG2 | GG3 | GG4 | GG5 | BW1 | BW2 | BW3 | BW4 | BW5 |  |
| OTU_1554 | *Brevibacterium* | 0 | 0 | 0 | 0 | 0.0081 | 0 | 0 | 0 | 0 | 0 | 1.039 |
| OTU_124 | *Brevundimonas* | 0.0356 | 0.0873 | 0.1479 | 0.0723 | 0.1358 | 0.0425 | 0.0153 | 0.0031 | 0.0055 | 0.0368 | 0.028 |
| OTU_436 | *Corynebacterium* | 0.0638 | 0.0030 | 0.0028 | 0.0060 | 0.0190 | 0.0025 | 0.0184 | 0.0281 | 0 | 0.0030 | 1.573 |
| OTU_518 | *Flavobacterium* | 0.0170 | 0.0214 | 0.0199 | 0.0241 | 0.0434 | 0.0326 | 0.0344 | 0 | 5.52E-03 | 0.0276 | 1.699 |
| OTU_900 | *Deinococcus* | 0.0127 | 0.0061 | 0 | 0.0090 | 0 | 0 | 0.0023 | 0 | 0.0027 | 0 | 0.344 |
| OTU_1273 | *Gordonia* | 0 | 0 | 0.0028 | 0 | 0.0027 | 0.0025 | 0 | 0 | 0 | 0.0031 | 2.989 |
| OTU_818 | *Mycobacterium* | 0.0085 | 0 | 0 | 0 | 0 | 0.0050 | 0 | 0 | 0 | 0 | 2.192 |
| OTU_855 | *Pseudomonas* | 1.1749 | 0.1504 | 0.3813 | 0.2380 | 0.5705 | 0.2361 | 0.2619 | 0.0375 | 0.0773 | 0.0645 | 0.26 |
| OTU_1763 | *Rhodococcus* | 0 | 0 | 0 | 0 | 0 | 0 | 0 | 0 | 0 | 0.0031 | 1.039 |
| OTU_30 | *Acinetobacter* | 0.4129 | 0.4682 | 0.1192 | 0.4385 | 0.0072 | 0.0038 | 0.3065 | 0.7748 | 0.0340 | 0.0056 | 2.711 |
| OTU_52 | *Sphingomonas* | 0.3448 | 0.3448 | 0.0185 | 0.3278 | 0.0681 | 0.2001 | 0.1192 | 0.2214 | 0.0127 | 0.3448 | 1.994 |

**Table S1**. The potential carotenoid producing bacteria predicted by PICRUSt.
